# Navigating the future of nano‐pesticides: A perspective on design, efficacy, mechanisms, and environmental stewardship

**DOI:** 10.1002/imt2.70129

**Published:** 2026-04-29

**Authors:** Xile Deng, Feiying Zhu, Heng Qiao, Wenjie Shangguan, Qin Yu, Sandeep Sharma, Vijayakumar Shanmugam, Yanan Deng, Yudan Wu, Pengyue Zhao, Ullah Farman, Lidong Cao, Shuo Yan, Zhichao Dong, Lianyang Bai

**Affiliations:** ^1^ Yuelushan Laboratory, Hunan Academy of Agricultural Sciences Changsha China; ^2^ Longping Agricultural College, Hunan University Changsha China; ^3^ College of Plant Protection, China Agricultural University Beijing China; ^4^ Institute of Plant Protection, Chinese Academy of Agricultural Sciences Beijing China; ^5^ The University of Western Australia Crawley Australia; ^6^ Institute of Nano Science and Technology Mohali India; ^7^ Department of Botany and Plant Sciences University of California Riverside California USA; ^8^ Xianghu Laboratory Hangzhou China; ^9^ Technical Institute of Physics and Chemistry, Chinese Academy of Sciences Beijing China

## Abstract

Nano‐pesticides are driving a paradigm shift toward sustainable plant protection. This review systematically synthesizes recent advances across four interconnected domains: (i) intelligent formulation design for targeted delivery and controlled release; (ii) interfacial behavior regulation to enhance foliar deposition; (iii) multi‐omics elucidation of synergistic efficacy and molecular interactions; and (iv) environmental fate management, including risk mitigation and microbiome remediation. By integrating these multiscale innovations, the field is advancing nano‐enabled crop protection from laboratory research toward field application. Collectively, this convergence provides a scientific foundation and interdisciplinary roadmap needed to build next‐generation agricultural systems that are resource‐efficient and ecologically compatible.

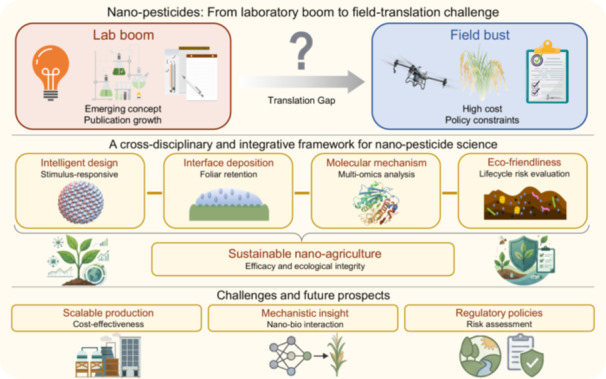

To the Editor,

Global agricultural systems face mounting challenges in achieving sustainability. Pesticides prevent the loss of an estimated 40% of global crop production annually to pests, diseases, and weeds, saving the global economy over US$220 billion [1]. While pesticides remain indispensable for crop protection, their field efficiency is often low: an estimated 10−75% of applied active ingredients (AIs) are lost through off‐target effects, such as runoff, leaching, volatilization, and spray drift, leading to ecological contamination, biodiversity decline, and accelerated resistance evolution [2]. This systemic inefficiency underscores the urgent need for strategies that reconcile productivity with ecological integrity [3]. Nano‐enabled agriculture, operating at the plant‐pesticide‐environment interface, offers a transformative alternative. Through smart delivery systems engineered for stimulus‐responsive release, targeted delivery and enhanced stability, and other capabilities, nanotechnology can improve pesticide utilization efficiency by approximately 30% while reducing off‐target impacts [4].

However, a critical question remains: Are nano‐pesticides a genuine breakthrough—or merely a formulation upgrade dressed in nanoscale novelty? Despite over a decade of research, their translation from lab to field remains erratic, hindered not by a lack of efficacy, but by insufficient mechanistic foresight and regulatory incoherence. This perspective, therefore, argues that the future of nano‐pesticides hinges not on smaller particles, but on smarter systems thinking, an approach articulated through a cross‐disciplinary framework that integrates principles from materials science, physics, biology, and environmental engineering. Such a framework represents a fundamental leap toward sustainable crop protection, offering a rational design paradigm that bridges efficacy with environmental compatibility. This perspective critically examines integrated multifunctional nanotechnologies through four interlinked dimensions: (i) intelligent design, which focuses on stimulus‐responsive carriers for controlled release; (ii) interface‐mediated enhancement, crucial for ensuring effective foliar deposition and retention; (iii) synergistic mechanism, employing multi‐omics tools to decipher molecular‐level efficacy; and (iv) environmental safety, which serves as the foundational assessment for sustainable deployment. To translate this potential into practice, these dimensions form a cohesive framework: intelligent design enables controlled delivery, interfacial deposition ensures field application, mechanistic insights guide targeted optimization, and environmental safety acts as the overarching constraint. Our central objective is to guide the development of nano‐enabled strategies that strengthen sustainable agroecosystem resilience, while safeguarding ecological integrity through proactive risk assessment and rigorous regulatory control.

## CLASSIFICATION AND STIMULUS‐RESPONSIVE FORMULATION STRATEGIES OF NANO‐PESTICIDES

**Figure 1:**
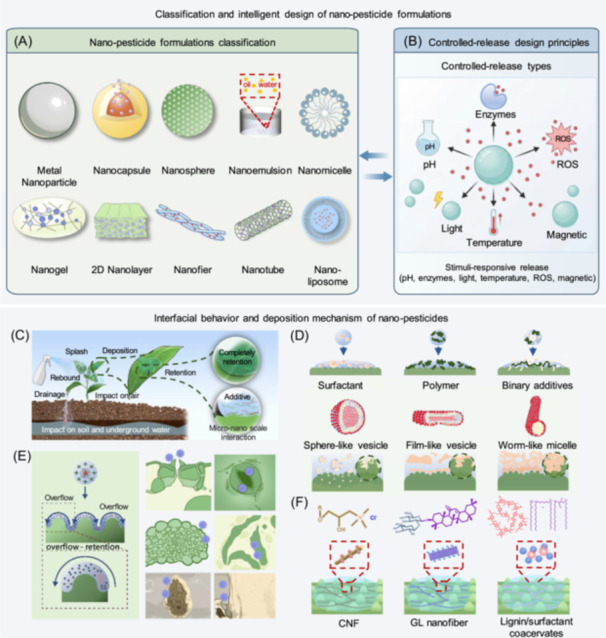
Classification and intelligent design of nano‐pesticide formulations. (A) Nano‐pesticides existing in the forms of metal nanoparticles, nanocapsules, nanospheres, nanoemulsions, nanomicelles, nanogels, 2D nanolayers, nanofibers, nanotubes, and nanoliposomes prepared by polymer carriers or small molecules through physical adsorption, encapsulation, coating, self‐assembly, etc. (B) Intelligent design of nano‐pesticides responsive to specific environmental signals (pH, enzyme activity, light, temperature, reactive oxygen species, and magnetic levels, etc.). Interfacial behavior and deposition (retention) mechanisms of nano‐pesticides. (C) Schematic outcomes of spray‐droplet impact on crop leaves, including spreading—deposition and retention versus rebound, splash, and drainage/runoff, which can lead to off‐target losses to air, soil, and groundwater. (D) Overflow‐retention on textured, waxy foliar surfaces: during impact and retraction, liquid locally overflows micro/nanostructures and becomes pinned, promoting micro‐nano‐scale accumulation of AIs/additives and formation of a more stable retained layer. (E) Representative microscopic observations illustrating the distribution of nano‐carriers/additives (blue balls) on the leaf surface and in near‐surface structures (e.g., epidermis/cuticle), highlighting their roles in deposition, adhesion, and subsequent uptake. (F) Additive‐enabled interfacial control strategies: surfactants, polymers, and binary formulations regulate dynamic surface tension, contact‐line pinning, and impact hydrodynamics through distinct self‐assembled morphologies (sphere‐like vesicles, film‐like vesicles, and worm‐like micelles), thereby suppressing rebound/splash and enhancing deposition efficiency; biomass‐derived nano‐additives (e.g., CNF, GL, and lignin/surfactant coacervates) further strengthen energy dissipation and adhesion networks to improve foliar retention and wash‐off resistance. CNF, cellulose nanofiber; GL nanofiber, glycyrrhizic acid nanofiber; ROS, reactive oxygen species; 2D Nanolayer, two‐dimensional nanolayer.

Compared to conventional pesticide formulations, nano‐pesticides offer distinct advantages, including, but not limited to, enhanced efficacy and bioavailability, controlled and targeted release, reduced application rates and environmental burden, as well as improved leaf adhesion with reduced drift. However, a globally recognized standard for the specific particle size range of nano‐pesticides has yet to be established. The agricultural industry standard of China, titled “Rules for drafting of specifications for nano‐pesticides product,” delineates the specific particle size ranges for various formulations. According to this standard, nano‐emulsions are defined within a 1−100 nm range, while both nano‐suspensions and nano‐water‐dispersible granules fall within a broader 1−300 nm range. Despite the lack of uniform size standards, a general international consensus has emerged on the classification of nano‐pesticides into two primary categories [5]. Type I includes nanomaterials with inherent pesticidal activity, such as Zn, Ag, Cu, Ti, and their corresponding oxides or sulfides. Type II comprises carrier‐based nano‐pesticides, in which AIs are incorporated using carriers via physical adsorption, chemical bonding, encapsulation, coating, self‐assembly, or other methods. Owing to the diversity in both carrier materials and fabrication methods, researchers have developed a broad spectrum of nano‐pesticides exhibiting a variety of morphological forms, including nanocapsules, nanospheres, nanoemulsions, nanomicelles, nanogels, 2D nanolayers, nanofibers, nanotubes, and nanoliposomes (Figure 1A) [6].

An ideal nano‐pesticide maintains optimal AI concentration over the required period to achieve effective pest control, while minimizing the crop residues and environmental persistence. Importantly, nanocarriers are not merely passive vehicles; they are fundamentally engineered towards achieving stimulus‐responsive intelligent release. In this paradigm, the AIs can be encapsulated within “smart” nanostructures responsive to specific environmental triggers, such as pH, enzymes, light, temperature, and reactive oxygen species (ROS) levels (Figure 1B) [7]. However, one must ask: do these “smart” releases truly respond to field‐relevant signals, or are they optimized under idealized lab conditions that rarely reflect the complexity of real agroecosystems? The practical translation of stimulus‐responsive nano‐pesticides from concept to field application depends on achieving precise spatiotemporal release, a key technological breakthrough that addresses the core limitations of conventional pesticides. Yet realizing this value is entirely contingent on proven field performance. Consequently, rational design must balance enhanced efficacy with new risk prevention, focusing on technical readiness and scalable production [8].

## INTERFACIAL ACTIVITY AND NANO‐ADJUVANTS FOR NANO‐PESTICIDES ON FOLIAR SURFACES

The effectiveness of stimulus‐responsive nano‐pesticides depends on their successful deposition onto target surfaces, especially leaves. A key but often overlooked factor affecting field performance is the millisecond‐scale physics of droplet impact on leaves [9]. Foliar surfaces are rarely ideal solids; they have multiscale curvature, veins, trichomes, and waxy textures that trap air and cause rebound or splashing of pesticides, especially on hydrophobic leaves (Figure 1C) [10]. Early impact dynamics determine the maximum AI retention, penetration, and action. Recent studies show deposition improvement relies on both equilibrium wettability and dynamic interfacial properties during droplet impact, as the air–liquid–solid interface forms and breaks rapidly [11]. Surfactants are key to interfacial control as their dynamic surface tension and adsorption kinetics govern spreading. Fast surfactants reduce the capillary barrier, speed contact‐line motion, and increase pinning, suppressing recoil and splashing (Figure 1D). The mesoscale organization of surfactants can change impact outcomes: vesicle‐forming or lamellar surfactants may restructure under deformation, enabling quick wettability on textured leaves, while traditional micellar formulations are less effective when an air cushion remains at the interface (Figure 1E). Polymeric adjuvants stabilize the lamella by increasing viscosity and dissipating impact energy, delaying rim breakup and suppressing satellite droplets [12]. Excess polymer loading may harm coverage uniformity. This prompts surfactant‐polymer designs that blend quick interfacial tension relaxation with viscoelastic damping, ensuring broad spreading and strong retention. Similarly, biomass‐derived nano‐adjuvants like cellulose, lignin complexes, and coacervates create interfacial networks that dissipate impact energy, mechanically interlock with leaf microstructures, enhance contact‐line pinning and wash‐off resistance, and reduce high surfactant use (Figure 1F). Emerging evidence indicates that leaf microtopography may actively influence droplet behavior via mechanosensing, an underexplored interface where biophysics interacts with plant physiology. Future nano‐adjuvants could lock into leaf textures biologically, not just physically. Integrating impact‐phase interfacial design—covering surfactant kinetics, polymer response, and bio‐nano network pinning—with nano carriers for controlled pesticide release creates a unified deposit‐retain‐penetrate framework. This strategy optimizes deposition and release profiles to boost pesticide efficiency and reduce off‐target loss.

## MULTI‐OMICS‐BASED SYNERGISTIC MECHANISMS FOR NANO‐PESTICIDES

**Figure 2:**
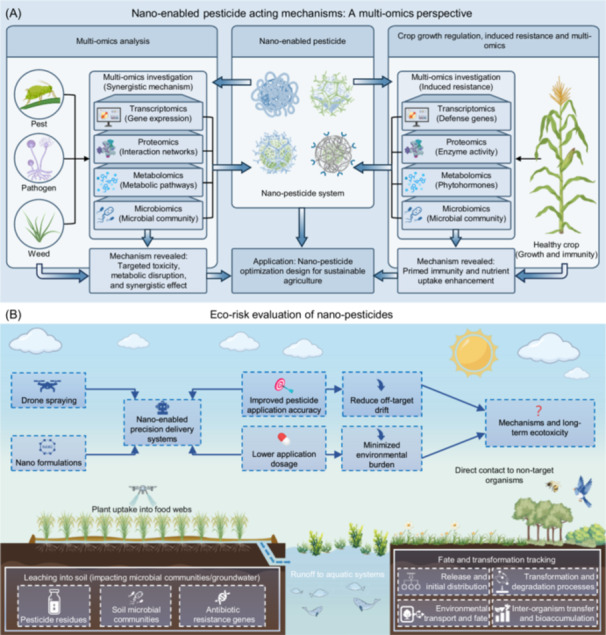
Multi‐omics mechanism of action and a forward‐looking framework for the evaluation of long‐term ecological risks of nano‐pesticides. (A) The network diagram of the action mechanism of nano‐pesticides by multi‐omics. (B) This schematic delineates a proactive framework for assessing the long‐term ecological risks of nano‐pesticides. Such a prospective evaluation is critical to identify and mitigate potential environmental hazards. Ultimately, it is indispensable for guiding the sustainable development of nano‐agrochemicals within a safe operating space.

The synergistic effect of stimulus‐responsive nano‐pesticides arises from a complex molecular process initiated by their interfacial deposition and subsequent penetration. These processes involve quaternary interactions among nano‐pesticides, target pests, host crops, and associated microorganisms. Compared with traditional methods, recent advances in transcriptomics, proteomics, metabolomics, and microbiome profiling technologies have enabled deeper elucidation of their multi‐target regulatory mechanisms from a biological perspective, thereby providing a paradigm shift over traditional methods of nano‐pesticides preparation and application. Taken together, integrated multi‐omics analyses indicate that such synergy primarily manifests along two dimensions: direct enhancement of pest toxicity and indirect induction of crop immune responses (Figure 2A).

In terms of pest control, integrated multi‐omics analyses have revealed mechanisms by which nanocarriers synergistically enhance toxicity through promoting exogenous substance uptake and disrupting host immune metabolism. For instance, researchers developed a double‐stranded ribonucleic acid (dsRNA) nano‐pesticide (dsRNA@ZIF‐8@PDA) targeting the fall armyworm (*Spodoptera frugiperda*). 16S rRNA sequencing analysis showed that this nano‐preparation could not only reduce the relative abundance of dominant *Enterococcus*, but also enrich pathogenic *Serratia*. Transcriptomics further identified endocytosis, phagosome, and immune deficiency and Toll pathways as crucial synergistic targets. Altering these pathways facilitates the cellular uptake of dsRNA, while the resulting gut dysbiosis suppresses the host's ROS immune response, thereby significantly amplifying the insecticidal efficacy of the RNA pesticide [13].

For crop resistance induction, multi‐omics integration analysis has revealed the synergistic immune mechanisms of nanomaterials: upregulation of defense‐related genes, modulation of hormone signals, and reshaping rhizosphere microbial communities. For example, transcriptome analysis showed that the nano‐protectant lentinan/star polycation complex markedly up‐regulated the expression of pathogenesis resistance (*Pathogenesis‐Related Protein 1*, *Pathogenesis‐Related Protein 10*, and *Osmotin‐Like Protein*) and antioxidant enzymes (*Catalase* and *Superoxide Dismutase*) related genes, thereby enhancing the resistance of tobacco seedlings to viruses [14]. Combined transcriptome and metabolome analysis further showed that the nano‐protectant diblock polymer–chitosan complex considerably enhanced the phenylpropanoid biosynthesis, increased the content of antibacterial secondary metabolites, and activated the plant hormone signal transduction pathway, which achieved the long‐term prevention and control of mango anthracnose [15]. Proteomics combined with metabolomics analysis showed that nanoscale boron nitride mitigated protein synthesis and folding disorder caused by *Fusarium* infection, and up‐regulated glutathione metabolism to eliminate the active oxygen. Additionally, nanoscale boron nitride also enhanced crop disease resistance indirectly by increasing soil humus and improving the rhizosphere micro‐ecology [16]. While multi‐omics has unraveled these molecular cascades, most current studies remain correlative, presenting a “black box” regarding causality. Thus, the next frontier requires establishing rigorous causal inference through synthetic biology. Collectively, multi‐omics integration has revealed the complex network of regulatory mechanisms of nano‐pesticides: directly targeting toxicity, inducing host resistance, and regulating symbiotic microorganisms. These mechanistic insights inform the rational design principles of stimulus‐responsive nanocarriers by identifying actionable targets to achieve synergistic efficacy.

## ENVIRONMENTAL FATE AND RISK ASSESSMENT FRAMEWORK FOR NANO‐PESTICIDES

Beyond the pursuit of efficacy, the ultimate sustainability of nano‐pesticides is governed by their environmental safety, a dimension that constrains and informs the rational design, application, and mechanistic understanding outlined in the preceding sections. Advanced nano designs (e.g., core‐shell) enable the controlled release of AIs, thereby mitigating both acute and chronic exposure risks to non‐target organisms, including pollinators, soil fauna, and aquatic species [17]. However, the distinctive physicochemical properties of nanomaterials, such as their high surface‐area‐to‐volume ratio and dynamic interfacial behavior, introduce new environmental complexities. Addressing these challenges, therefore, requires a proactive, scenario‐based safety assessment framework spanning the entire technological lifecycle. Built on a mechanistic understanding of environmental fate, such a framework can employ scenario‐driven modeling to identify critical exposure pathways, including leaching into soil (impacting microbial communities and groundwater), runoff into aquatic systems, plant uptake into food webs, and direct contact with vulnerable species.

Following field application, nano‐pesticides undergo environmental aging processes such as dissolution, aggregation, and surface transformations resulting from interactions with natural organic matter and microbial exudates, ultimately leading to the formation of an “eco‐corona.” If the eco‐corona dictates the fate, bioavailability, and toxicity of nanomaterials, can we intentionally engineer surface properties to recruit beneficial coronas—those that promote degradation, reduce toxicity, or even support soil health? This “corona‐by‐design” approach could transform risk into opportunity, highlighting the critical importance of understanding and actively steering these corona formation processes. These transformations critically determine the environmental persistence, mobility, and bioavailability of nano‐pesticides. The resulting alteration in physicochemical properties governs their transport within soil‑water systems and profoundly influences their behavior across food webs. Through plant uptake or contaminated water, nano‐pesticides and their transformation products can enter non‑target organisms, including beneficial insects, birds, and fish, thereby initiating trophic transfer and potential bioaccumulation. Potentially posing risks to ecosystem integrity and food‑web safety. Although precision delivery technologies, such as drone‑assisted spraying of nano‑formulations, are intended to reduce off‑target exposure, particularly for indicator species (e.g., birds, fishes, and bees), significant knowledge gaps remain. At present, the molecular mechanisms underlying the observed reduction in toxicity of nano‐pesticides are not fully elucidated. Similarly, the long‑term ecotoxicological effects on soil microbial communities and their ecological functions, as well as the potential influence of nano‐pesticides on the evolution and dissemination of antibiotic resistance genes, remain insufficiently understood. Equally concerning is the limited research addressing chronic low‑dose exposure and transgenerational effects in non‑target organisms, whether through dietary or environmental pathways [18]. Therefore, systematically elucidating the long‑term environmental fate and ecological impacts of nano‑pesticides is essential. Future priorities for proactive governance should include: (i) a “corona‐by‐design” approach to favor less toxic eco‑coronas; (ii) integrated scenario‑based lifecycle modeling with precision delivery to minimize off‑target exposure; and (iii) systematic research on chronic, transgenerational, and microbiome‑related effects, including antibiotic resistance (Figure 2B).

## PROSPECTS AND CHALLENGES FOR NANO‐PESTICIDES

This review provides a systematic analysis and integrated roadmap that identifies cost, scalability, and safety as the key translational barriers for nanopesticides, and proposes convergent solutions across design, application, and regulation. Building on this framework, the widespread application of nano‐pesticides is limited by several challenges, including high production costs, scalable manufacturing constraints, and unresolved safety concerns regarding carrier materials [19]. For instance, Kah et al. note that a minimum 20% improvement in efficacy, productivity, or cost is needed for broader market access [2]. Overcoming these barriers requires integrated solutions: developing low‐cost, eco‐friendly natural carriers; establishing scalable platforms such as microfluidics; and utilizing machine learning for rational carrier‐AI design. Underpinning all these efforts, however, is the need for deeper mechanistic insight. Future efforts should prioritize three frontiers: (i) optimizing droplet behavior via leaf microstructure and surfactant systems to enhance deposition; (ii) elucidating post‐entry biological responses, such as intracellular trafficking and nanomaterial‐induced effects (e.g., oxidative stress); and (iii) advancing environmental and mammalian toxicology of carriers, focusing on their fate, ecological effects, and long‐term safety. Research in these areas is essential for predicting efficacy, assessing risks, and designing next‐generation nano‐pesticides.

Concurrently, establishing a robust and adaptive regulatory framework is critical to steer this innovation responsibly. However, there is currently no dedicated international framework for the registration and management of nano‐pesticides. Instead, they are regulated under existing frameworks for chemicals, which may include provisions for nanomaterials. The European Union evaluates the nanoscale components of pesticides under the Registration, Evaluation, Authorization, and Restriction of Chemicals regulations. In the United States, while nanoformulations typically follow the same legal framework as conventional products, those exhibiting novel properties attributable to the nanoscale form may be subject to additional review under the Toxic Substances Control Act. China is at the forefront of nano‐pesticides regulation, having issued the world's first official technical standard for such products [20]. These examples reflect worldwide efforts to adapt regulatory frameworks to address nanotechnology in agriculture, with most regions tending to adapt existing frameworks or adopt case‐by‐case assessment approaches.

Unlocking the transformative potential of nano‐pesticides hinges on a convergent strategy that synergizes technological innovation, fundamental scientific insight, and proactive regulatory oversight. This necessitates a shift from conventional chemical‐centric models to nano‐specific governance systems, featuring specialized testing standards, clear legal definitions for nano‐forms, and harmonized international safety protocols. Strengthening the registration process by mandating nano‐specific characterization and lifecycle assessment as prerequisites for market approval will be critical to safeguarding both innovation and public safety. Ultimately, this trajectory of nano‐pesticides will be shaped not only by scientific ingenuity but also by the global governance frameworks we build today. We therefore call for the establishment of an open, international consortium to co‐develop standards, share toxicological data, and align regulatory definitions. Without such coordination, we risk fragmenting innovation and repeating the mistakes of past agrochemical revolutions.

## AUTHOR CONTRIBUTIONS

**Xile Deng**: Conceptualization; investigation; funding acquisition; writing—original draft; methodology; validation; visualization; writing—review and editing; software; formal analysis; project administration; data curation; supervision; resources. **Feiying Zhu**: Investigation; methodology; validation; software; formal analysis; visualization; writing—review and editing. **Heng Qiao**: Investigation; validation; methodology; data curation; software. **Wenjie Shangguan**: Investigation; methodology; formal analysis; data curation; validation. **Qin Yu**: Investigation; methodology; validation; formal analysis; data curation; writing—review and editing. **Sandeep Sharma**: Writing—review and editing. **Vijayakumar Shanmugam**: Writing—review and editing. **Yanan Deng**: Writing—review and editing. **Yudan Wu**: Writing—review and editing. **Pengyue Zhao**: Writing—review and editing. **Ullah Farman**: Writing—review and editing. **Lidong Cao**: Supervision; conceptualization; writing—original draft; writing—review and editing. **Shuo Yan**: Conceptualization; writing—original draft; writing—review and editing; supervision. **Zhichao Dong**: Conceptualization; writing—original draft; writing—review and editing; supervision. **Lianyang Bai**: Conceptualization; writing—original draft; writing—review and editing; supervision. All authors have read the final manuscript and approved it for publication.

## CONFLICT OF INTEREST STATEMENT

The authors declare no conflicts of interest.

## ETHICS STATEMENT

No animals or humans were involved in this study.

## Data Availability

The data that support the findings of this study are openly available in [repository name] at [DOI]. No new data or code were generated or analyzed in this study. Supplementary materials (graphical abstract, slides, videos, Chinese translated version, and updated materials) may be found in the online DOI or iMeta Science http://www.imeta.science/.
